# Effect of Polymer and Crosslinker Concentration on Static and Dynamic Gelation Behavior of Phenolic Resin Hydrogel

**DOI:** 10.3390/gels10050325

**Published:** 2024-05-09

**Authors:** Wenjuan Ji, Bei Chang, Haiyang Yu, Yilin Li, Weiqiang Song

**Affiliations:** 1 School of Petroleum Engineering, China University of Petroleum (East China), Qingdao 266580, China; jwj1224@126.com; 2 Chuanqing Drilling Engineering Company Limited, China National Petroleum Corporation Limited, Chengdu 610051, China; changb_cqyyq@cnpc.com.cn; 3 College of Energy and Mining Engineering, Shandong University of Science and Technology, Qingdao 266590, China; skd996020@sdust.edu.cn; 4 Drilling & Production Technology Research Institute of Jidong Oilfield, China National Petroleum Corporation Limited, Tangshan 063000, China; zcy_liyl@petrochina.com.cn

**Keywords:** PFR hydrogel, dynamic gelation, gelation time, partially hydrolyzed polyacrylamide, plugging ability

## Abstract

The application results of profile control and water plugging technology are highly related to the gelation time and strength of phenolic resin hydrogel. In this work, a hydrogel solution was prepared by fully mixing the prepared polymer solution with a crosslinker. The static gelation process of PFR hydrogel in ampoule bottles and porous media was analyzed by changes in the viscosity and residual resistance coefficient. Then, the dynamic gelation of the PFR hydrogel in porous media was tested using a circulating flow device, and the changes in viscosity and injection pressure were analyzed during the dynamic gelation process. Finally, the effects of the polymer concentration and crosslinker concentration on dynamic gelation were analyzed. The initial gelation time and final gelation time in porous media were 1–1.5 times and 1.5–2 times those in ampoule bottles under static conditions, respectively. The initial dynamic gelation time in porous media was 2–2.5 times and 1.5–2 times the initial static gelation times in ampoule bottles and porous media, respectively. The final dynamic gelation time was four times and two times the initial static gelation times in ampoule bottles and porous media, respectively. The production after dynamic gelation in porous media comprised hydrogel aggregates and water fluid, leading to a high injection pressure and low viscosity of the produced liquid. As the concentration of polymer and crosslinker increased, the dynamic gelation time was shortened and the gel strength was increased. In the dynamic gelation process in porous media, the phenol resin hydrogel could migrate deeply, but it was limited by the concentrations of the polymer and crosslinker. The results of subsequent water flooding showed that the polymer hydrogel had a good plugging ability after dynamic gelation. The deep reservoir could only be blocked off in the subsequent water flooding process when the migration of hydrogel happened in the dynamic gelation process.

## 1. Introduction

Polymer hydrogel is widely used as a profile control agent in worldwide oil fields [1,2,3,4], and its application results are highly related to gelation time and gel strength. The gelation time is divided into static gelation time and dynamic gelation time. Many polymer hydrogels can form whole structures with adjustable gelation times and controllable strength on the ground. However, it is difficult to predict the gelation situation after injection into the formation, which leads to application problems in profile control and water plugging [5]. A field application case showed that polymer hydrogel injected into a deep formation would not show an obvious blockage after extending the static gelation time by 20 days. When subjected to shear in the rock core, whether the polymer gelation solution can form hydrogel or not is related to the dynamic gelation of polymer hydrogels. This directly affects the success of profile control and water plugging.

Recently, many scholars have studied the differences between the dynamic and static gelation of hydrogel. Ki et al. studied the fractal nature of the backbone network for the irreversible kinetic gelation model. They suggested that the static percolation model might be adequate to describe the sol–gel transition and related phenomena of the irreversible growth model [6]. Dolan et al. described the gelation time and strength of the hydrogel using small-amplitude oscillation under zero shear rate and stable shear conditions [7]. Kolnes et al. studied the effects of shear and temperature on the gelation of xanthan gum/chromium hydrogel, and the results showed that shear disrupted the cross-linking structure of the polymer and crosslinker [8]. Bhaskar et al. investigated the effect of different shear modes on the gelation process of redox systems [9]. Josset et al. studied the gelation process of cross-linked polymer hydrogel under a high shear rate and high shear conditions in the formation near the wellbore [8]. Carvalho et al. studied the cross-linking reaction process of cross-linked polymers during the shearing process. The results showed that shearing could promote or delay the cross-linking reaction with a critical shear rate, switching between these two effects [10]. McCool et al. studied the gelation behavior of chromium acetate weak hydrogel in porous media flow using a 1036 feet long stainless-steel conduit [11]. Series et al. used a 100 feet long thin iron pipe to simulate the flow of hydrogel in cracks. The results showed that the residual resistance coefficient reached its peak at 20 feet and then began to decrease, while the middle and rear ends (60–100 feet) of the thin iron pipe remained unchanged [12]. Abete et al. studied the structure and dynamics in the formation of irreversible gels by molecular dynamics simulations, and suggested that gelation transition was due to the random percolation of permanent bonds between neighboring particles [13]. Sun, Z. et al. studied the influences of polymer molecular weight and concentration on gelation time, gel strength, and adhesion ability, and indicated that increasing the molecular weight and concentration could shorten the gelation time and enhance the gel strength, and that adhesion strength is mainly related to the number of hydrogen bonds [14]. Khurshid, I. and Afgan, I. analyzed the effect of polymer drive composition on surfactant retention and new surface complexation reactions [15]. Our research group has also studied the dynamic gelling behavior of polymer hydrogels in recent years. The static gelation time and the main factors of the gel strength of hydrogel at high temperatures were studied, resulting in the conclusion that temperature is an important factor affecting the gelation process of hydrogel [16]. The effect of the injected speed on dynamic gelation was analyzed in reference [17], showing that the injection speed has little effect on the dynamic gelation time, but has a great effect on the gel strength. Mechanical shear rate can also have an important influence on the gelling behavior of hydrogels [18]. The static gelation and dynamic gelation of hydrogels are quite different due to many factors, such as adsorption and shear [19]. The results showed that the dynamic gelling behavior of polymer hydrogels was greatly affected by the shear rate, which was directly related to the reservoir permeability and injection rate.

Previous studies have clarified the static and dynamic gelation processes of hydrogel. However, the migration characteristics and main control factors after dynamic gelation in porous media are not clear, especially the influence of the mass concentration of hydrogel on the migration characteristics. When the polymer hydrogel migrates in the formation, it is subjected to core shear and its structure is destroyed. During the migration process, it forms a whole hydrogel or a dispersed hydrogel particle structure. What are the changes in the gelation time and gel strength in formation and on the ground? These issues directly affect the success of profile control and water plugging by polymer hydrogel. Therefore, it is of great importance to study the gelation time and gel strength during the dynamic gelation process of polymer hydrogels [20,21]. Based on the above issues, this article studied static gelation in ampoule bottles and porous media, and the dynamic gelation of a hydrogel system composed of ordinary partially hydrolyzed polyacrylamide HPAM and phenol formaldehyde resin (PFR). The relationship between gelation time and gel strength under these three different conditions was investigated, and a quantitative relationship between the static gelation time and dynamic gelation time was established. The plugging ability of static gelation and dynamic gelation in porous media for subsequent water flooding was comparatively analyzed.

## 2. Results and Discussion

### 2.1. Static Gelation in Ampoules

**Figure 1:**
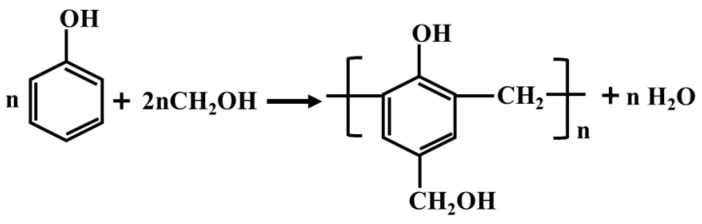
Reaction mechanism of water-soluble phenolic resin crosslinker.

**Figure 2:**
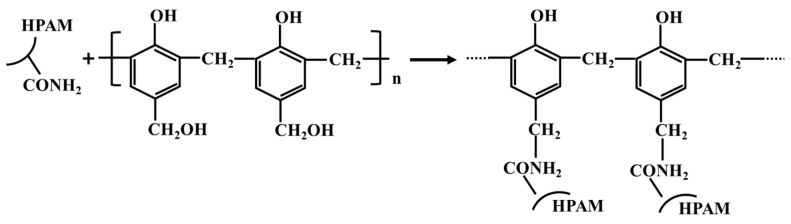
Formation mechanism of the bulk gel.

A water-soluble phenolic resin crosslinker was obtained by the poly-condensation of phenol and formaldehyde (excess) under the catalyst action of sodium hydroxide [22]. The reaction mechanism of the crosslinker is shown in Figure 1. The hydroxymethyl groups (-CH_2_OH) on the crosslinker and the amide groups (-CONH_2_) on the polymer were dehydrated and condensed to form a bulk gel. The formation mechanism of the bulk gel is shown in Figure 2.

**Figure 3:**
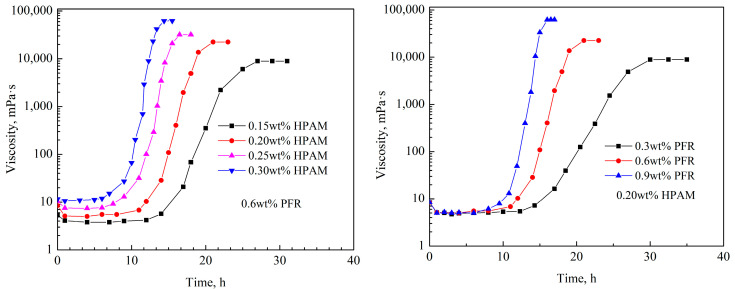
The change in viscosity of PFR hydrogel with time.

**Figure 4:**
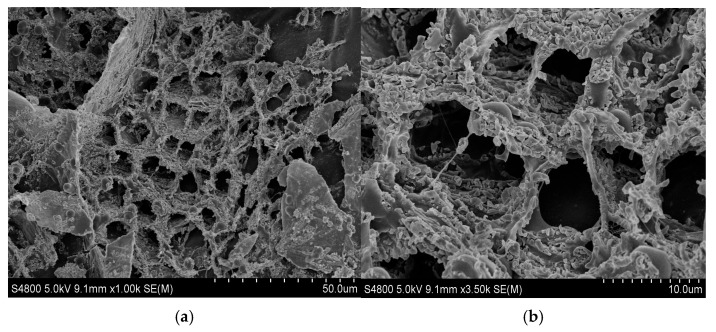
The micro morphology of PFR hydrogel after static gelation in ampoule bottles; (**a**) micro morphology at 50 μm; (**b**) micro morphology at 10 μm.

The commonly used methods for determining the gelation time of hydrogel include the visual strength code method and viscosity method [23,24]. This article used the viscosity method to determine the relationship between the viscosity of the hydrogel system and time under different gelation times (see Figure 3). It could be seen that the viscosity did not change significantly with time before the cross-linking reaction began. During the gelation process, the viscosity rapidly increased with time and then stabilized [18]. The probability of contact between the amide groups (-CONH_2_) and hydroxymethyl groups (-CH_2_OH) in the system increased with increasing concentrations of polymer and crosslinker, which made the formed spatial network structure denser. The gelation time of the hydrogel was shortened and the gel strength was increased [19]. According to the research results of Mokhtari et al., the gelation time can be divided into the initial gelation time (IGT) and final gelation time (FGT). The initial gelation time refers to the moment when the viscosity of the system begins to significantly increase at the beginning of the cross-linking reaction. The final gelation time is the time when the system viscosity reaches stability at the end of the cross-linking reaction [25]. The initial gelation time and final gelation time with different formulations of hydrogels can be obtained from the curves in Figure 3. The micro morphology of the PFR hydrogel after static gelation in ampoule bottles is shown in Figure 4.

As shown in Figure 4, the microscopic morphology of the phenolic resin hydrogel can be clearly seen at a lower magnification, and the phenolic resin hydrogel formed a regular network structure after static gelation in the ampoule bottle. The regular network structure was formed by the cross-linking of the amide groups in the polymers with the hydroxymethyl groups in the phenolic resin prepolymers. At higher multiples, it was observed that there were pores of similar size in the middle of the network structure, with pore sizes distributed around 3–5 μm.

### 2.2. Static Gelation in Porous Media

**Figure 5:**
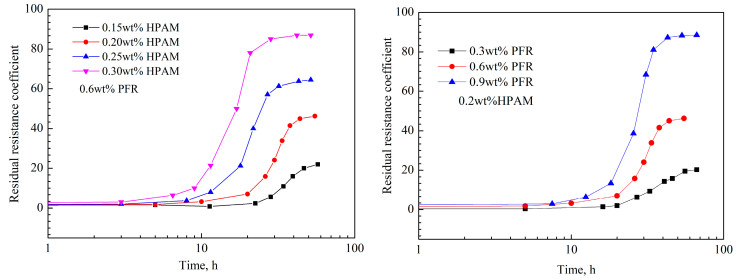
The change in static gelation of PFR hydrogel with time in porous media.

The amide groups on the polymer and the hydroxymethyl groups on the crosslinker could be cross-linked to form a three-dimensional network structure, which had a certain plugging ability on porous media. The strength of three-dimensional network structure was related to the concentration of the hydrogel, which directly affected the plugging efficiency of the porous media. The pressure change characteristics of the cross-linked polymer hydrogel when flowing in porous media reflect the degree of the hydrogel cross-linking reaction. As the cross-linking reaction proceeds, the plugging ability of the hydrogel for porous media gradually increases until the end of the reaction [17]. Generally, indicators such as the resistance coefficient and residual resistance coefficient or transition pressure are used to describe the flow characteristics of cross-linked polymer hydrogel [26,27]. The residual resistance coefficient is calculated by measuring the breakthrough pressure gradient of hydrogel in a series of sand pack pipes to characterize the process of the cross-linking reaction of hydrogel. The values reflect the viscosity of the hydrogel in porous media (see Figure 5).

**Figure 6:**
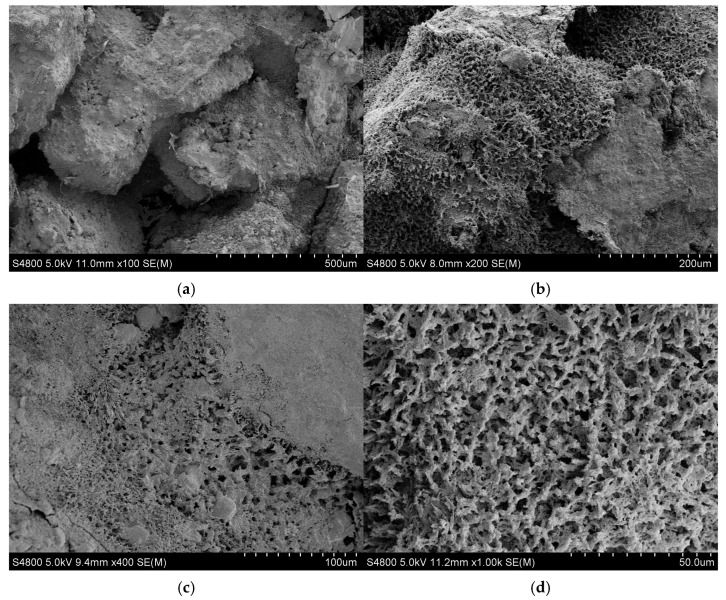
The micro morphology of PFR hydrogel after static gelation in porous media (**a**) micro morphology at 500 μm; (**b**) micro morphology at 200 μm; (**c**) micro morphology at 100 μm; (**d**) micro morphology at 50 μm.

From Figure 5, it can be seen that the residual resistance coefficient first showed no significant change with an increasing placement time, and then rapidly increased until it stabilized. The reaction process of hydrogel can be divided into three stages: induction, gelation, and stabilization. The initial gelation time and final gelation time were both shortened with increasing concentrations of polymer and crosslinker. The micro morphology of the PFR hydrogel after static gelation in porous media is shown in Figure 6.

As shown in Figure 6, the existence form of phenolic resin hydrogel after static gelation in porous media can be clearly reflected at a lower magnification. The hydrogels were mainly adsorbed on the surface of porous media and trapped at smaller pore throats. At a higher magnification, it can be observed that the structure of phenolic resin hydrogel was composed of a network structure formed by the winding and cementation of some thicker chains and larger pores. Under the action of shear failure and adsorption retention, the phenolic resin hydrogel network structure formed in the process of static gelation in porous media was not as clear and complete as that formed by static gelation in ampoule bottles.

The results of the comparison of static gelation in ampoule bottles and porous media are shown in Table 1. During the hydrogel injection into the sandpack pipes, the polymer and crosslinker were subjected by the shear of porous media, which led to prolonging the gelation time and reducing the gel strength. Due to the different molecular sizes of the polymer and crosslinker, there was a significant difference in their migration speed in the porous media. A change in the concentration ratio of the polymer and crosslinker can affect the gelation time. The adsorption of the polymer and crosslinker on the surface of the porous media directly led to a decrease in the number of molecules involved in the cross-linking reaction, thereby prolonging the gelation time [28]. Therefore, the static gelation time of the PFR hydrogel in porous media was longer than that in ampoules, with an initial gelation time of 1–1.5 times and a final gelation time of 1.5–2 times.

### 2.3. Dynamic Gelation in Porous Media

#### 2.3.1. Analysis of Dynamic Gelation Process

**Figure 7:**
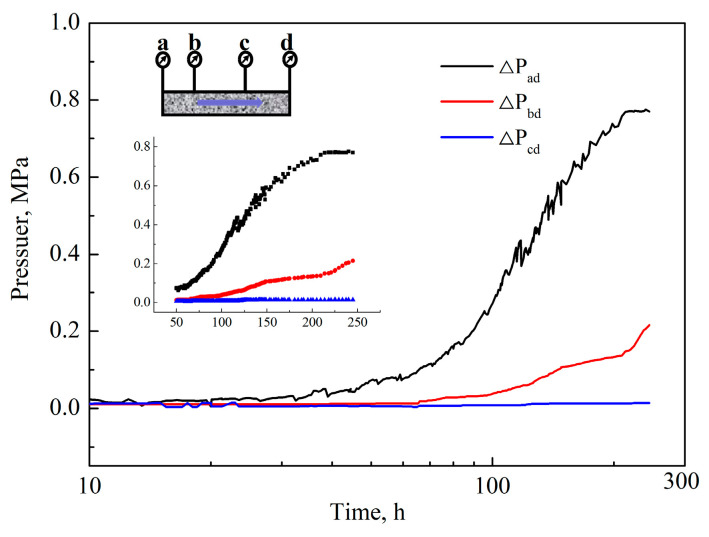
The change in pressure of PFR hydrogel with time in the dynamic gelation process.

In the static gelation of the hydrogel in porous media, there was no shear force affecting the cross-linking process, ultimately forming a three-dimensional network structure. However, the hydrogel could be subjected to the shear force from the porous media in the dynamic gelation process. When the hydrogel network structure size was increased to larger than the pore size, the network structure could be destroyed by the shear force, forming dispersed hydrogel particles rather than a whole bulk hydrogel. The phenolic resin hydrogel 0.2 wt% HPAM+0.3 wt% PFR was analyzed using a circulating flow experimental device. The change in the injection pressure (ΔP_ad_, ΔP_bd_, and ΔP_cd_) was recorded with time (see Figure 7). During the dynamic gelation process, ΔP_ad_ is the pressure difference between the injection end a and the outlet end d, ΔP_bd_ is the pressure difference between the measuring point b and the outlet end d, and ΔP_cd_ is the pressure difference between the measuring point b and the outlet end d. The permeability was 7.07 μm^2^ and the injected speed was 0.5 mL/min.

**Figure 8:**
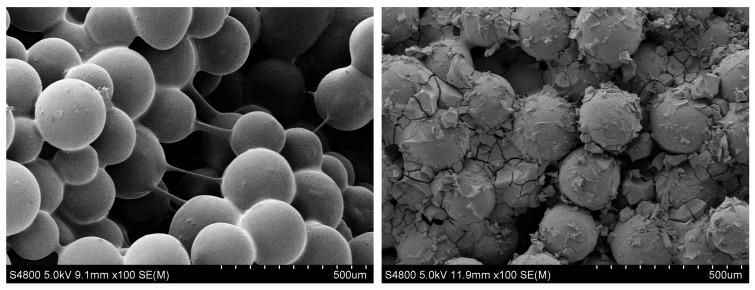
The micro morphology of PFR gel after dynamic gelation in porous media.

From Figure 7, in the dynamic gelation process, the pressure difference ΔP_ad_ firstly changed smoothly, then rapidly increased before finally stabilizing, which indicates that the hydrogel underwent the induction stage, gelation stage, and stabilization stage. During the induction stage, the polymers that had not been cross-linked still maintained a certain degree of viscoelasticity. When passing through a smaller core throat, they were compressed and deformed to generate a certain degree of elasticity, which required a certain pressure difference to pass through [29]. At this stage, the polymer molecules still formed as individual particles rather than a network structure [30]. Macroscopically, there was no significant change in pressure. During the gelation stage, polymer hydrogels were subjected by two types of forces: cross-linking reaction force and shear fragmentation force. The former could enhance the three-dimensional network structure of the hydrogel and increase the apparent viscosity, while the latter could destroy the network structure and reduce the apparent viscosity. When the size of the phenolic resin hydrogel increased with time until its cohesion was overcome by the shear of the porous media, the hydrogel was sheared and destroyed to form a dispersed gel particle system, instead of becoming a whole hydrogel, as shown in Figure 8. The formed hydrogel particles remained at the pore throat of the porous media to play a plugging role, thus leading to the subsequent pressure rise.

The microstructure of the phenolic resin hydrogel after dynamic gelation in porous media was dispersed hydrogel particles rather than a whole bulk hydrogel. The network structure of the phenolic resin hydrogel was not found at a higher magnification. These hydrogel particles were adsorbed on the surface of the porous media and aggregated at the pore throat, reducing the seepage capacity of the porous media and playing a plugging role. Compared to the static gelation of the phenolic resin hydrogel in porous media, the main form of the hydrogel particles after dynamic gelation was trapping. The viscosity results of the hydrogel solution from the middle container after the experiment are shown in Table 2. It could be seen that, after the dynamic gelation process in the porous media, the viscosity of the hydrogel solution was lower than the viscosity of the initial polymer gelation solution. This confirmed that the polymer was cross-linked, but hydrogel particles were formed under the shear action of the porous media. The adsorption and migration of the hydrogel particles in the porous medium kept the displacement pressure gradually increasing. The output liquid was free water, which was generated by the bound water inside the hydrogel under the action of shear force. The pressure difference ΔP_bd_ saw a small increase, and the start time of this increase was significantly delayed compared to the pressure difference ΔP_ad_. This shows that hydrogel particles formed by dynamic gelation can realize deep migration in porous media.

#### 2.3.2. The Effect of Polymer and Crosslinker Concentration on Dynamic Gelation

**Figure 9:**
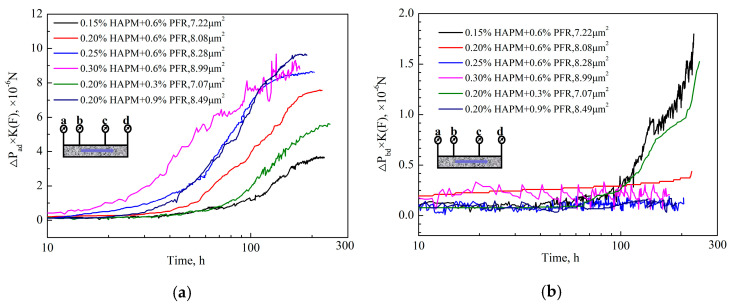
The change in dynamic gelation of PFR hydrogel with time in porous media under different concentrations of polymer and crosslinker; (**a**) ΔP_ad_ × K vs. time; (**b**) ΔP_bd_ × K vs. time.

The dynamic gelation experiments of the PFR hydrogel in porous media under different formulations were carried out by changing the concentrations of the polymer and crosslinker. The injection rate of 0.5 mL/min was kept unchanged (see Figure 9). Due to the small change in the pressure difference ΔP_bd_ and almost no change in the pressure difference ΔP_cd_, the dynamic gelation process was characterized by the pressure difference ΔP_ad_. The dynamic initial gelation time of PFR hydrogel in porous media refers to the time that the cross-linking reaction begins, and viscosity increases significantly during the flow process [31]. This is shown in the figure as the first inflection point on the inlet pressure difference curve ΔP_ad_. The final gelation time refers to the moment when the inlet pressure difference ΔP_ad_ no longer increases and tends to stabilize, which is expressed as the second inflection point on the inlet pressure difference ΔP_ad_ curve. In order to eliminate the influence of permeability, F = ΔP × K is defined as the seepage resistance of fluid flowing in a porous medium.

The cross-linking active points increased with increasing concentrations of polymer and crosslinker. Under the same shear conditions, the cross-linking reaction rate accelerated and the gelation time was shortened. Therefore, the time for the inflection point to appear on the inlet pressure difference curve was advanced, indicating that the initial gelation time and final gelation time of the dynamic gelation in porous media were both shortened (Figure 9a). From Figure 9b, it can be seen that, as the concentrations of polymer and crosslinker agent increased, especially to a polymer concentration greater than 0.2 wt%, the change amplitude of ΔP_bd_ × K decreased. This indicates that the migration ability of the PFR hydrogel during dynamic gelation was limited by the concentrations of the polymer and crosslinker. According to the experimental method of dynamic gelation in porous media, it can be seen that the polymer hydrogel solution with two times the pore volume (2 PV) flowed alternately in the porous media during the entire cycle. When 1 PV hydrogel solution flowed in the porous media at 75 °C, the other PV hydrogel solution flowed in the intermediate container at room temperature (25 °C). The hydrogel solution of 0.2 wt% HPAM+0.6wt% with an initial viscosity value of 8.4 mPa·s aged at room temperature for 30 days. The viscosity was 10.9 mPa·s, indicating that the PFR hydrogel solution was not cross-linked at room temperature. Therefore, the dynamic gelation time in porous media should be half the time required for the entire gelation process. The initial and final gelation time were obtained through dynamic gelation experiments in porous media with different polymer and crosslinker concentrations, and were compared with static gelation in ampoule bottles and porous media. The IGTs and FGTs of the PFR hydrogel in the process of dynamic gelation are shown in Table 3.

According to Table 3, the initial and final gelation times of dynamic gelation in PFR hydrogel porous media were longer than those in ampoule bottles and porous media. The dynamic initial gelation time was 2–2.5 times the static initial gelation time in ampoule bottles and 1.5–2 times the static initial gelation time in porous media. According to Formula (1), the shear rate of the PFR hydrogel during dynamic gelation in porous media is calculated [32,33,34], as shown in Table 4.

(1) $$\gamma =\frac{3n+1}{n}\cdot \frac{v}{\sqrt[{0.5}]{8C\prime K\phi }}$$

*γ*—shear rate, s^−1^; n—viscosity index of fluid, mPa·s^n^; *v*—injection rate, cm/s; *C*’—the coefficient related to tortuosity, usually ranging from 25/12 to 2.5; *K*—permeability, μm^2^; and *Φ*—porosity. The porosity value is the ratio of the saturated water volume to the total volume, and the tortuosity value is the average of the lower and upper limits.

According to Table 4, there was a certain shear rate during the dynamic gelation process of the PFR hydrogel in porous media. There were two forces in the process of PFR hydrogel formation: one was the cross-linking effect that facilitated the formation of the network structure, and the other was the shear effect that destroyed the network structure. Therefore, the initial gelation time and final gelation time of dynamic gelation in porous media were longer than those of static gelation in ampoule bottles and porous media.

#### 2.3.3. Analysis of Water Flooding after Dynamic Gelation

**Figure 10:**
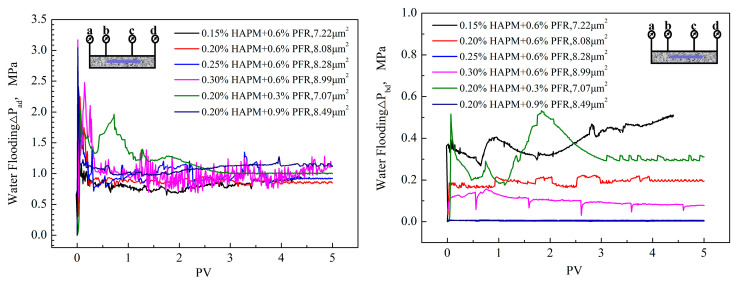
The change in water flooding after dynamic gelation with pore volume under different concentrations of polymer and crosslinker.

After the dynamic gelation process of the phenolic resin hydrogel was completed, the changes in the pressure difference of subsequent water flooding were recorded at each point with the injection water volume (see Figure 10). According to the experimental method of the dynamic gelation of phenolic resin hydrogel, it can be seen that, during the entire dynamic gelation process, there were two PV phenolic resin hydrogel solutions circulating alternately in the porous media. Therefore, the residual resistance coefficient of the subsequent water flooding was generated by the two PV phenolic resin hydrogels.

From Figure 10, it can be seen that, under different concentrations of polymer and crosslinker, the subsequent water flooding pressure difference ΔP_ad_ exhibited the same change trend after the dynamic gelation of the phenolic resin hydrogel. With an increase in the water flooding pore volume, ΔP_ad_ first rapidly increased to the maximum value, then decreased and stabilized. The change in ΔP_bd_ was related to the concentrations of the polymer and crosslinker. When the concentrations of the polymer and crosslinker were relatively small, ΔP_bd_ showed a certain value, especially if the polymer concentration was less than 0.2wt%. When the pressure difference ΔP_bd_ in the process of dynamic gelation had a certain value, the subsequent water flooding pressure difference ΔP_bd_ showed significant changes. This indicates that only when the phenolic resin hydrogel migrated during the dynamic gelation process would it have a plugging effect on the deep formation during subsequent water flooding. The residual resistance coefficient of water flooding after dynamic gelation in porous media is calculated, as shown in Table 5.

According to Table 5, the residual resistance coefficient of the ad section gradually increased with increasing concentrations of polymer and crosslinker. Increases in the numbers of polymer and crosslinker molecules were conducive to the formation of a high-strength hydrogel, which was shown by the increase in the plugging strength of the porous media. Therefore, the residual resistance coefficient of subsequent water flooding increased. The difference was not significant compared with the residual resistance coefficient of water flooding after static gelation in porous media, indicating that the phenolic resin hydrogel after dynamic gelation had a good plugging ability. The residual resistance coefficient of the ad section decreased with increasing concentrations of polymer and crosslinker. When the concentration of the polymer was greater than 0.25 wt% and the concentration of the crosslinker was greater than 0.9 wt%, effective plugging could not be formed after the dynamic gelation of the bd section. The concentrations of polymer and crosslinker were positively correlated with the gel strength. The storage modulus and loss modulus of the hydrogel increased with increasing concentrations of polymer and crosslinker, which made the hydrogel difficult to deform and gave it a strong resistance to impact and local damage. Hydrogel with a high internal friction resistance finds it difficult to migrate in rock pores. Therefore, hydrogel with a high gel strength was mainly retained at the injection end after dynamic gel formation, which cannot produce effective plugging in deep formations.

## 3. Conclusions

The field implementation of hydrogel deep profile control can greatly improve oil recovery. It is affected by the dynamic gelation of hydrogel in porous media, especially the influence of the mass concentration of hydrogel components on the migration rule after dynamic gelation. This paper studied the quantitative relationship between dynamic and static gelling, the regulation of the subsequent water flooding of dynamic gelation, and the influence of the polymer concentration and crosslinker concentration on the deep migration of hydrogel. The main conclusions were as follows:

(1) The quantitative relationship between the static gelation of hydrogels in different environments was established. The static gelation time of PFR hydrogel in porous media was longer than that in ampoule bottles, where the initial gelation time increased to 1~1.5 times and the final gelation time increased to 1.5~2 times, respectively. The phenolic resin hydrogel could form a three-dimensional network structure after static gelation in ampoule bottles and porous media.

(2) The quantitative relationship between the dynamic gelation of hydrogels in different environments was established, and revealed the existing form of hydrogels after dynamic gelation. The dynamic initial and final gelation time of PFR hydrogel in porous media was longer than the static initial gelation time in ampoule bottles and porous media. The phenolic resin hydrogel could only form dispersed hydrogel particles after dynamic gelation in porous media.

(3) The subsequent water flooding experiment of dynamic gelation showed that the residual resistance coefficient of water flooding was not significantly different from that after static gelation in porous media, indicating that the polymer hydrogel after dynamic gelation had a good plugging ability.

(4) The phenolic resin hydrogel could realize deep migration during dynamic gelation in porous media, but it was limited by the concentrations of the polymer and crosslinker. The deep reservoir could only be blocked off in the subsequent water flooding process when the migration of the hydrogel happened in the dynamic gelation process.

## 4. Materials and Methods

### 4.1. Materials

The polymer employed in this research was the ordinary partially hydrolyzed polyacrylamide (HPAM), whose molecular weight was 1.2 × 10^7^ and the degree of hydrolysis was 22%. The crosslinker was a water-soluble phenolic resin prepolymer, which was obtained by the poly-condensation of phenol and formaldehyde (excess) under the catalyst action of sodium hydroxide [20]. The used synthetic water (SW) contained 6921 ppm Na^+^, 412 ppm Ca^2+^, 148 ppm Mg^2+^, and 11853 ppm Cl^−^.

### 4.2. Experimental Methods

#### 4.2.1. Static Gelation in Ampoule Bottles

The viscosity method was used to determine the relationship between the viscosity of the hydrogel system and time under different gelation times. The experimental method was as follows: the prepared polymer solutions were diluted with simulated water, then crosslinker agents with different concentrations were added. After thorough mixing, they were placed in a constant temperature oven at 75 °C. The viscosity of the system was measured at different gelation times using a DV-II viscometer.

#### 4.2.2. Static gelation in Porous Media

The permeability and pore volume of sandpack pipe models (Φ 2.5 cm × 10 cm) were measured after being saturated with water. In total, 1 PV of hydrogel solution was injected into every pipe, and the sandpack pipe models were placed in a 75 °C oven. At each interval, a sandpack pipe was taken out to carry out the water flooding experiment. The relationship between the pressure and pore volume was measured and the residual resistance coefficient was calculated under 1 mL/min during water flooding. The determination of the gelation time was due to the relationship between the residual resistance coefficient and gelling time.

#### 4.2.3. Dynamic Gelation in Porous Media

**Figure 11:**
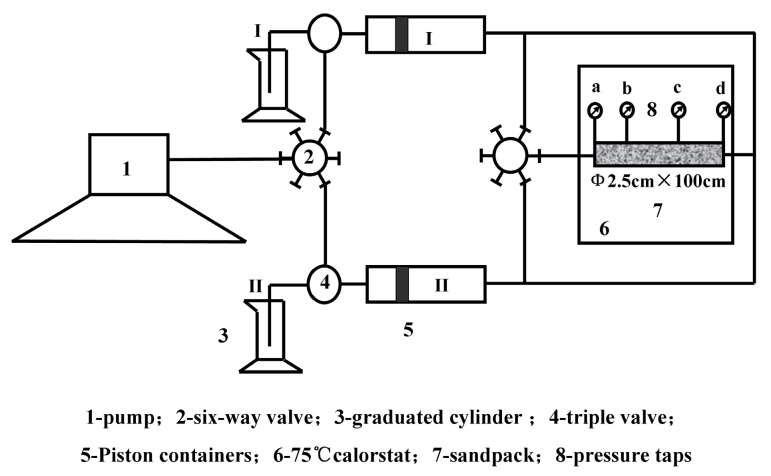
Schematic of circulated equipment for dynamic gelation in porous media.

The dynamic gelation of HPAM/PFR hydrogel in porous media could be estimated with the circulating device, which comprised two piston containers at room temperature and a sandpack at 75 °C. Specific methods have been shown in the literature [15], as shown in Figure 11.

#### 4.2.4. Water Flooding after Dynamic Gelation in Porous Media

In order to confirm the residual resistance coefficient of the used hydrogel system after dynamic gelation, subsequent water flooding under 1 mL/min was conducted when the process of dynamic gelation was finished. The plugging ability could be determined by the curve of the pressure difference with the injected pore volume of water.

## Figures and Tables

**Table 1:** The IGTs and FGTs of PFR hydrogel in the process of static gelation in ampoule bottles and porous media.

No	HPAM, %	PFR, %	Static Gelation in Ampoule Bottles		Static Gelation in Porous Media	
			IGT, h	FGT, h	IGT, h	FGT, h
1	0.15	0.6	14	27	25	45
2	0.2	0.6	12	21	17	40
3	0.25	0.6	9	16.5	10	29
4	0.3	0.6	7	14.4	8	23
5	0.2	0.3	14.3	30	20	45
6	0.2	0.9	9.5	18	10	35

**Table 2:** Viscosity of PFR hydrogel production after dynamic gelation in porous media.

HPAM, wt%	PFR, wt%	Speed, mL/min	K, μm^2^	Viscosity, mPa‧s
0.15	0.6	0.5	7.22	2.1
0.2	0.6	0.5	8.08	3.7
0.25	0.6	0.5	8.28	6.3
0.3	0.6	0.5	8.99	4.2
0.2	0.3	0.5	7.07	5.7
0.2	0.9	0.5	8.49	1.6

**Table 3:** The IGTs and FGTs of PFR hydrogel in the process of dynamic gelation.

No	HPAM , %	PFR , %	Dynamic Gelation in Porous Media	
			IGT, h	FGT, h
1	0.15	0.6	40	105
2	0.2	0.6	25	95
3	0.25	0.6	20	80
4	0.3	0.6	14	46
5	0.2	0.3	35	106
6	0.2	0.9	20	80

**Table 4:** Shear rate of PFR hydrogel under the process of dynamic gelation in porous media.

HPAM, wt%	PFR , wt%	Speed, mL/min	K, μm^2^	Porosity	n	C’	Shear Rate, s^−1^
0.2	0.3	0.5	7.07	0.352	0.573	2.29	4.34
0.2	0.6	0.5	8.08	0.367	0.440	2.29	3.71
0.2	0.9	0.5	8.48	0.371	0.445	2.29	3.61
0.15	0.6	0.5	7.22	0.353	0.645	2.29	4.43
0.25	0.6	0.5	8.28	0.369	0.427	2.29	3.63
0.3	0.6	0.5	8.99	0.373	0.346	2.29	3.28

**Table 5:** The residual resistance coefficient of water flooding after dynamic gelation of hydrogel with different concentrations of polymer and crosslinker.

HPAM , wt%	PFR , wt%	K, μm^2^	Speed, mL/min	Residual Resistance Coefficient	
				ad	bd
0.15	0.6	7.22	0.5	98	77
0.2	0.6	8.08	0.5	101	33
0.25	0.6	8.28	0.5	112	-
0.3	0.6	8.99	0.5	150	3
0.2	0.3	7.07	0.5	104	46
0.2	0.9	8.49	0.5	140	-

## Data Availability

The data presented in this study are openly available in article.

## References

[1] Al-Muntasheri G.A., Nasr-El-Din H.A., Peters J.A., Zitha P.L.J. (2006). Investigation of a High-Temperature Organic Water-Shutoff Gel: Reaction Mechanisms. SPE J..

[2] Banerjee R., Patil K., Khilar K.C. (2006). Studies on Phenol-Formaldehyde Gel Formation at a High Temperature and at Different pH. Can. J. Chem. Eng..

[3] Aldhaheri M., Wei M.Z., Zhang N., Bai B.J. (2019). A Review of Field Oil-Production Response of Injection-Well Gel Treatments. SPE Res. Eval. Eng..

[4] Khurshid I., Afgan I. (2022). Geochemical Investigation of Electrical Conductivity and Electrical Double Layer Based Wettability Alteration During Engineered Water Injection in Carbonates. J. Petrol. Sci. Eng..

[5] Zhou B.B., Kang W.L., Yang H.B., Zhu T.Y., Zhang H.W., Li X.X., Sarsenbekuly B., Sarsenbek T. (2021). Preparation and Properties of an Acid-Resistant Preformed Particle Gel for Conformance Control. J. Petrol. Sci. Eng..

[6] Ki D.Y., Woo K.Y., Lee S.B. (2000). Static and Dynamic Properties of the Backbone Network for the Irreversible Kinetic Gelation Model. Phys. Rev. E.

[7] Dolan D.M., Thiele J.L., Willhite G.P. (1998). Effects of pH and Shear on the Gelation of a Cr(III)-Xanthan System. SPE Prod. Oper..

[8] Kolnes J., Stavland A., Thorsen S. The effect of temperature on the gelation time of xanthan/Cr(III) systems. Proceedings of the SPE International Conference on Oilfield Chemistry.

[9] Bhasker R., Stinson J., Willhite G., Thiele J. (1988). The effects of shear history on the gelation of polyacrylamide/chromium (VI)/thiourea solutions. SPE Reserv. Eng..

[10] Carvalho W., Djabourov M. (1997). Physical Gelation under Shear for Gelatin Gels. Rheol. Acta.

[11] McCool S., Li X.P., Willhite G.P. (2009). Flow of a Polyacrylamide/Chromium Acetate System in a Long Conduit. SPE J..

[12] Seright R. (1997). Use of Preformed Gels for Conformance Control in Fractured Systems. Old Prod. Facil..

[13] Abete T., De Candia A., Del Gado E., Fierro A., Coniglio A. (2007). Static and Dynamic Heterogeneities in a Model for Irreversible Gelation. Phys. Rev. Lett..

[14] Sun Z., Wang S., Zhu Q., Cao X., Lv K., Feng Y., Yin H. (2023). Insights into Polyacrylamide Hydrogels Used for Oil and Gas Exploration: Gelation Time, Gel Strength, and Adhesion Strength. Energy Fuels.

[15] Khurshid I., Afgan I. (2022). Novel insights into the geochemical evaluation of polymer drive composition on surfactant retention in carbonates using the surface complexation modeling. Sci. Rep..

[16] Yu H.Y., Jiang X.R., Ji W.J., Song W.Q., Cao Y.M., Yan F., Luo C., Yuan B. (2023). The New Low Viscosity and High-Temperature Resistant Composite Hydrogel. Chem. Pap..

[17] Yu H.Y., Ma Z.F., Tang L., Li Y.S., Shao X.Z., Tian Y.X., Qian J., Fu J., Li D., Wang L. (2022). The Effect of Shear Rate on Dynamic Gelation of Phenol Formaldehyde Resin Gel in Porous Media. Gels.

[18] Yu H.Y., Yu J.F., Ji W.J., Zheng J.P., Wang Y.F. (2021). Dynamic Gelation of the HPAM/Phenol-Formaldehyde Resin Gel under Oscillatory Shear: Critical Gelation Shear Rate and Reformation. Chem. Pap..

[19] Yu H.Y., Ji W.J., Zheng J.P. (2020). Dynamic and Static Gelation Behavior of Phenol Formaldehyde Resin Gel System in Ampoule Bottles and Porous Media. Oil Gas Sci. Technol..

[20] Albonico P., Bartosek M., Malandrino A., Bryant S., Lockhart T.P. Studies on Phenol-Formaldehyde Crosslinked Polymer Gels in Bulk and in Porous Media. Proceedings of the SPE International Symposium on Oilfield Chemistry.

[21] Bryant S.L., Rabaioli M.R., Lockhart T.P. (1996). Influence of Syneresis on Permeability Reduction by Polymer Gels. SPE Prod. Oper..

[22] Shi J., Wei X. (2003). Synthesis and Evaluation of Polymer Oil Displacement Agent Crosslinked by Phenolaldehyde Resin Prepolymer. Adv. Fine Petrochem..

[23] Sydansk R. A New Conformance-Improvement-Treatment Chromium(III) Gel Technology. Proceedings of the DOE Symposium on Enhanced Oil Recovery.

[24] Reddy B.R., Eoff L., Dalrymple E.D., Brown D. (2005). Natural Polymer-Based Compositions Designed for Use in Conformance Gel Systems. SPE J..

[25] Mokhtari M., Ozbayoglu M.E. Laboratory Investigation on Gelation Behavior of Xanthan Crosslinked with Borate Intended to Combat Lost Circulation. Proceedings of the SPE International Production and Operations Conference and Exhibition.

[26] Smith J.E. The Transition Pressure: A Quick Method for Quantifying Polyacrylamide Gel Strength. Proceedings of the SPE International Conference on Oilfield Chemistry.

[27] Prada A., Civan F., Dalrymple E.D. Evaluation of Gelation Systems for Conformance Control. Proceedings of the SPE Improved Oil Recovery Conference.

[28] Park P.J., Sung W. (1998). Polymer Translocation Induced by Adsorption. J. Chem. Phys..

[29] Delshad M., Kim D.H., Magbagbeola O.A., Huh C., Pope G., Tarahhom F. Mechanistic Interpretation and Utilization of Viscoelastic Behavior of Polymer Solutions for Improved Polymer-Flood Efficiency. Proceedings of the SPE Improved Oil Recovery Conference, SPE Improved Oil Recovery Conference.

[30] Chauveteau G., Tabary R., Renard M., Omari A. Controlling In-Situ Gelation of Polyacrylamides by Zirconium for Water Shutoff. Proceedings of the SPE International Symposium on Oilfield Chemistry.

[31] Yu H.Y., Wang Y.F., Zhang J., Lv P., Shi S.L. (2015). Dynamic Gelation of HPAM/Cr(III) under Shear in an Agitator and Porous Media. Oil Gas Sci. Technol..

[32] Savins J.G. (1969). Non-Newtonian Flow Through Porous Media. Ind. Eng. Chem..

[33] Hirasaki G.J., Pope G.A. (1974). Analysis of Factors Influencing Mobility and Adsorption in the Flow of Polymer Solution Through Porous Media. SPE J..

[34] Camilleri D., Engelson S., Lake L.W., Lin E.C., Ohno T., Pope A.G., Sepehrnoori K. (1987). Description of an Improved Compositional Micellar/Polymer Simulator. SPE Reserv. Eng..

